# Anomalous Origin of Left Main Coronary Artery from the Right Sinus of Valsalva: A Case Series-based Review

**DOI:** 10.7759/cureus.7777

**Published:** 2020-04-22

**Authors:** Muhammad S Khan, Owais Idris, Jay Shah, Ravina Sharma, Hemindermeet Singh

**Affiliations:** 1 Internal Medicine, Mercy St Vincent Medical Center, Toledo, USA; 2 Cardiology, Mercy St Vincent Medical Centre, Toledo, USA; 3 Internal Medicine, Mercy St Vincent Medical Centre, Toledo, USA; 4 Interventional Cardiology, Ascension St John Hospital and Medical Centre, Dretroit, USA

**Keywords:** coronary artery angiography, anomalous coronary artery, left main coronary artery, right coronary cusp, coronary artery bypass grafting

## Abstract

Congenital anomalies involving the origin of coronary arteries are rare and the most common anomaly is left circumflex (LCX) arising from the right sinus of Valsalva (RSV). Other anomalies include a single coronary artery from the left sinus of Valsalva, both coronary arteries from RSV and left anterior descending coronary artery (LAD) from RSV. Anomalous origin of left main from RSV carries a high risk of sudden cardiac arrest. A retrospective analysis and literature review of three patients admitted to our medical center with the acute coronary syndrome, who underwent coronary angiography and were found to have left main coronary artery (LMCA) originating from the right coronary cusp (RCA). One patient had non-diseased coronaries with symptoms caused by the variant anatomy with possible compression of the LMCA, whereas the other two patients had 100% occluded RCA with variable stenosis in the left coronary system. Eventual surgical re-implantation with bypass grafting was required in all three patients. LMCA from the RSV is a rare, but often fatal anomaly. Due to a lack of data and inability to predict sudden cardiac death, the latest guidelines recommend surgical intervention (class 1 recommendation) for all patients with LMCA from RSV, regardless of ischemia or ischemic symptoms.

## Introduction

Congenital anomalies of origin of coronary arteries are rare, with an incidence from 0.24% to 1.3% of the cases [1, 2]. The most critical anomaly is the left main coronary artery (LMCA) arising from the right sinus of Valsalva (RSV) as it has a high risk of sudden cardiac death [2]. The most common coronary artery anomaly is the left circumflex artery (LCX) arising from the RSV or right coronary artery (RCA). Other anomalies include a single coronary artery from the left sinus of Valsalva, both coronary arteries from RSV and left anterior descending coronary artery (LAD) from RSV [3]. We report a case series and literature review of three patients who presented with the acute coronary syndrome and were found to have anomalous LMCA arising from the RSV on angiogram.

## Case presentation

Case 1

**Figure 1:**
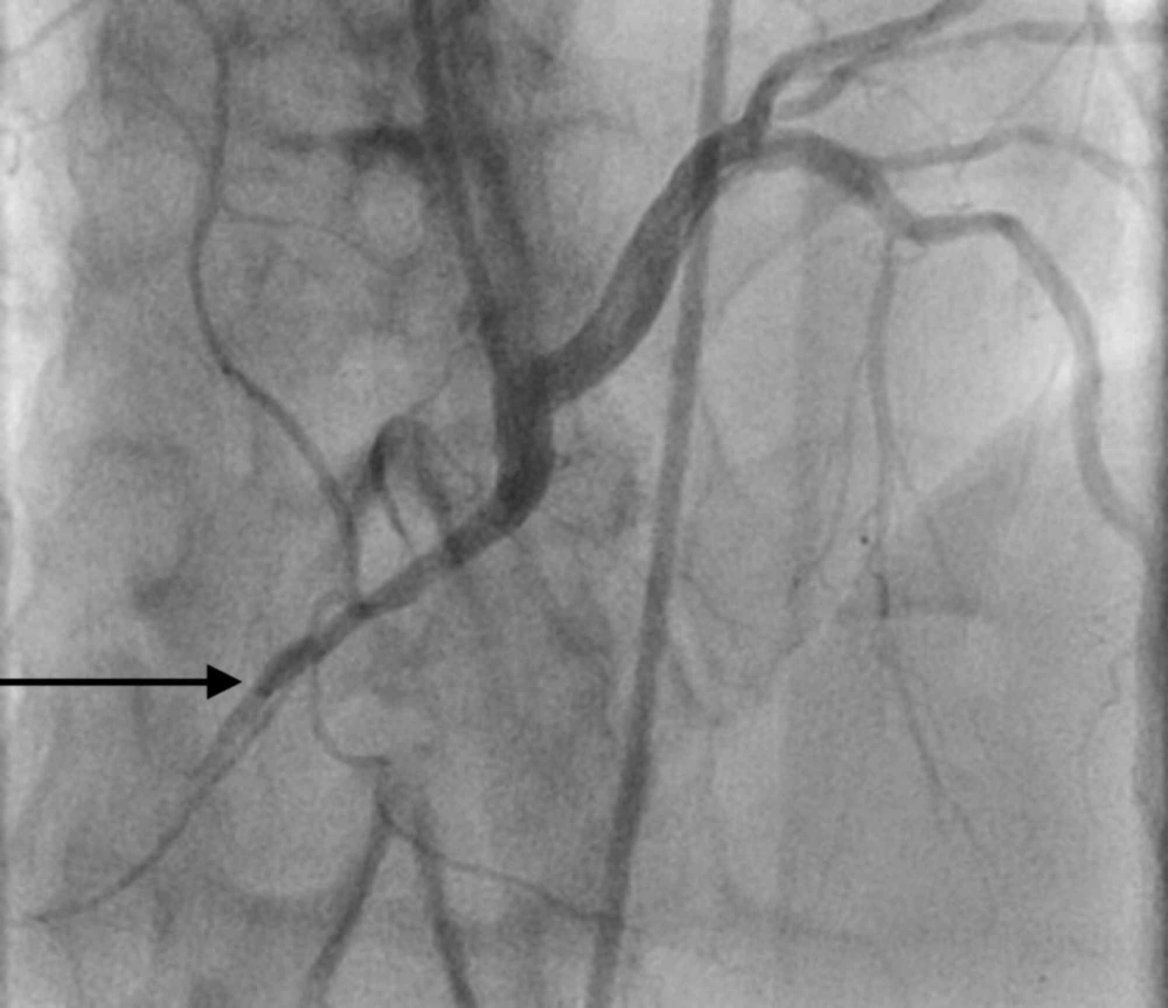
Selective coronary angiography of the right coronary artery revealing the common origin of the RCA and LMCA along with 100% stenosis in mid-RCA with TIMI-0 flow distally (arrow) RCA - right coronary cusp; LMCA - left main coronary artery; TIMI - thrombolysis in myocardial infarction

**Figure 2:**
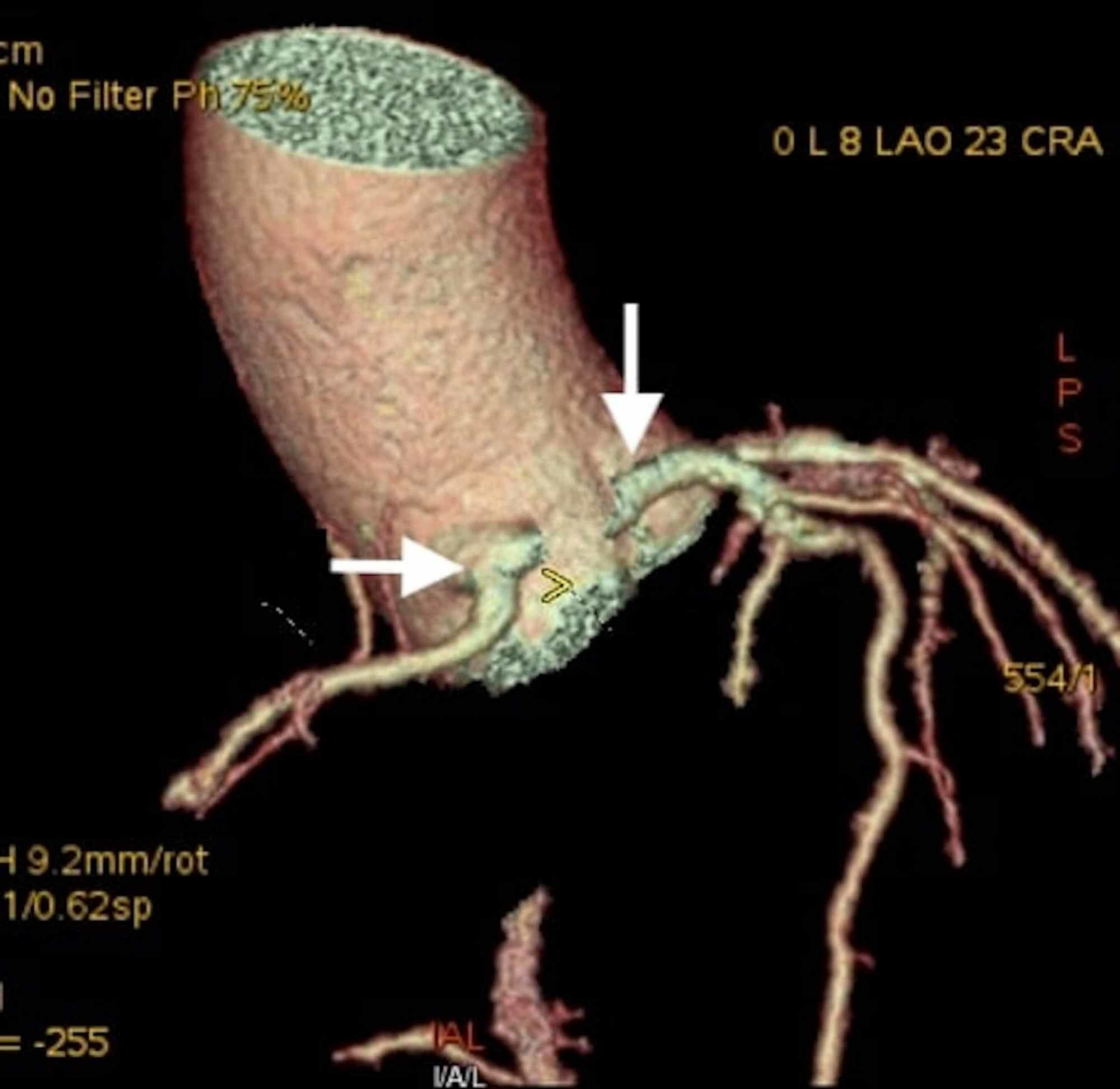
Coronary CTA image showing the RCA (horizontal white arrow) and LMCA (vertical white arrow) originating from the right coronary cusp CTA - computerized tomography angiogram; RCA - right coronary cusp; LMCA - left main coronary artery

A 78-year-old African American male presented to the emergency department (ED) complaining about nausea, vomiting, and substernal chest pain for one day. He was a chronic smoker and had a pertinent medical history of hypertension, hyperlipidemia, ischemic stroke, and peripheral vascular disease. Initial electrocardiogram (ECG) showed ST elevation in leads II and arteriovenous fistula (AVF), suggesting acute inferior infarct. The patient was hemodynamically stable, and his chest pain resolved after initial medical therapy. An urgent coronary angiogram as part of an early invasive strategy revealed an anomalous origin of LMCA from the right coronary cusp (see Figure 1). The RCA had 100% occlusion in mid-segment with left-to-right collaterals. A decision was thus made to proceed with a coronary computerized tomography angiogram (CTA) to rule out a malignant course. Coronary CTA confirmed an anomalous common trunk origin of right and left coronary artery from the RSV with an inter-arterial and intramural course of LMCA across the wall of the ascending aorta and behind the pulmonary artery (see Figure 2). Cardiothoracic surgery (CTS) consultation was obtained at this point, and coronary artery bypass grafting (CABG) was recommended due to the malignant course of left main and high-grade stenosis in RCA. The patient subsequently underwent quintuple bypass with the right internal mammary artery (RIMA) to LAD, left internal mammary artery (LIMA) to ramus, saphenous vein graft (SVG) to diagonal, obtuse marginal (OM) and RCA. He had an uneventful postoperative hospital course and was discharged without complications. It has been about three years since his CABG, and his last cardiac catheterization was performed about two years ago, showing patent grafts with minimal non-obstructive stenosis.

Case 2

**Figure 3:**
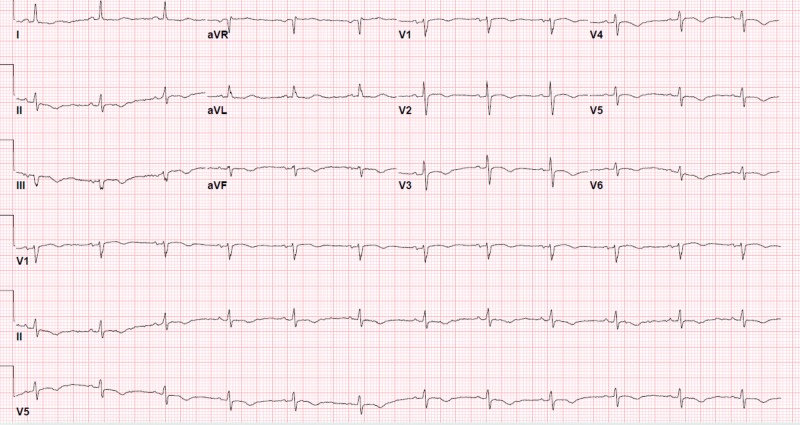
Initial Electrocardiogram showing non-specific T wave abnormality in the inferolateral leads

**Figure 4:**
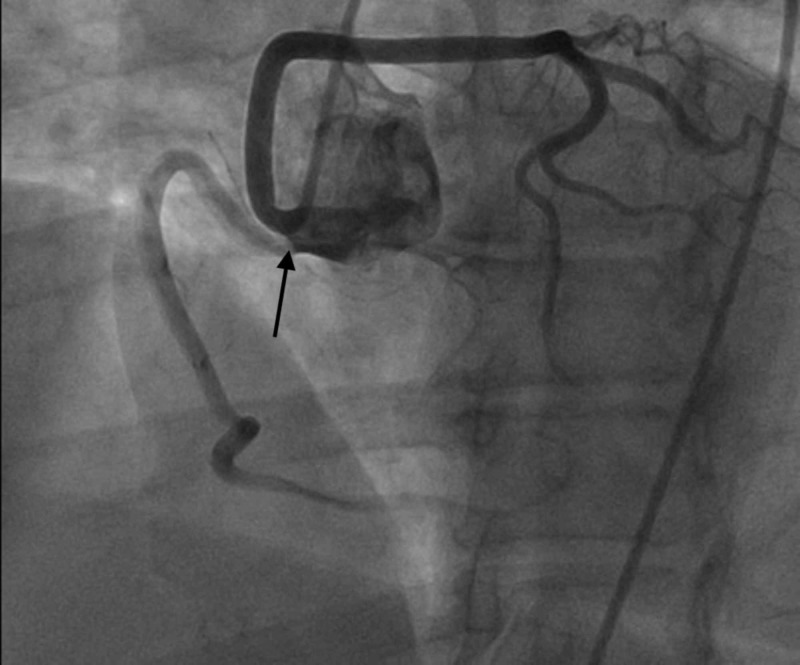
Selective coronary angiography of the right coronary artery revealing common ostia of the RCA (black arrow) without any obstructive disease of the coronary arteries and LMCA with TIMI-3 flow RCA - right coronary cusp; LMCA - left main coronary artery; TIMI - thrombolysis in myocardial infarction

A 47-year-old Hispanic female presented to ED with a two-day history of intermittent substernal chest pressure increasing in frequency and intensity. She had a significant family history of premature coronary artery disease (CAD), including her biological brother requiring CABG at 34 years of age. She also had a history of hypertension and was a chronic smoker. Her initial ECG showed a T wave abnormality in the inferolateral leads (Figure 3). Subsequently, cardiac catheterization was performed for unstable angina, which revealed an anomalous origin of LMCA from the RSV with common ostia of the RCA and LMCA. She did not have any obstructive disease in the coronary arteries per se (Figure 4). A decision was made to obtain coronary CTA to delineate the course of the left main further; however, the patient started having active chest pain with worsening of ECG changes post cardiac catheterization. CTS was consulted, and the patient was taken emergently to the operating room. Intraoperatively, the LMCA was noted to be originating from the extreme right side of the RSV with an intramural course in its proximal segment, a likely cause of compression of LMCA ostium leading to myocardial ischemia and given presentation. The LMCA was successfully re-implanted on the anterior surface of the aorta. She had an unremarkable postoperative hospital course and was discharged four days after. She was apparently re-admitted one week later for a large pericardial effusion requiring drainage and eventual pericardial window. On follow-up, she has continued to do well.

Case 3

**Figure 5:**
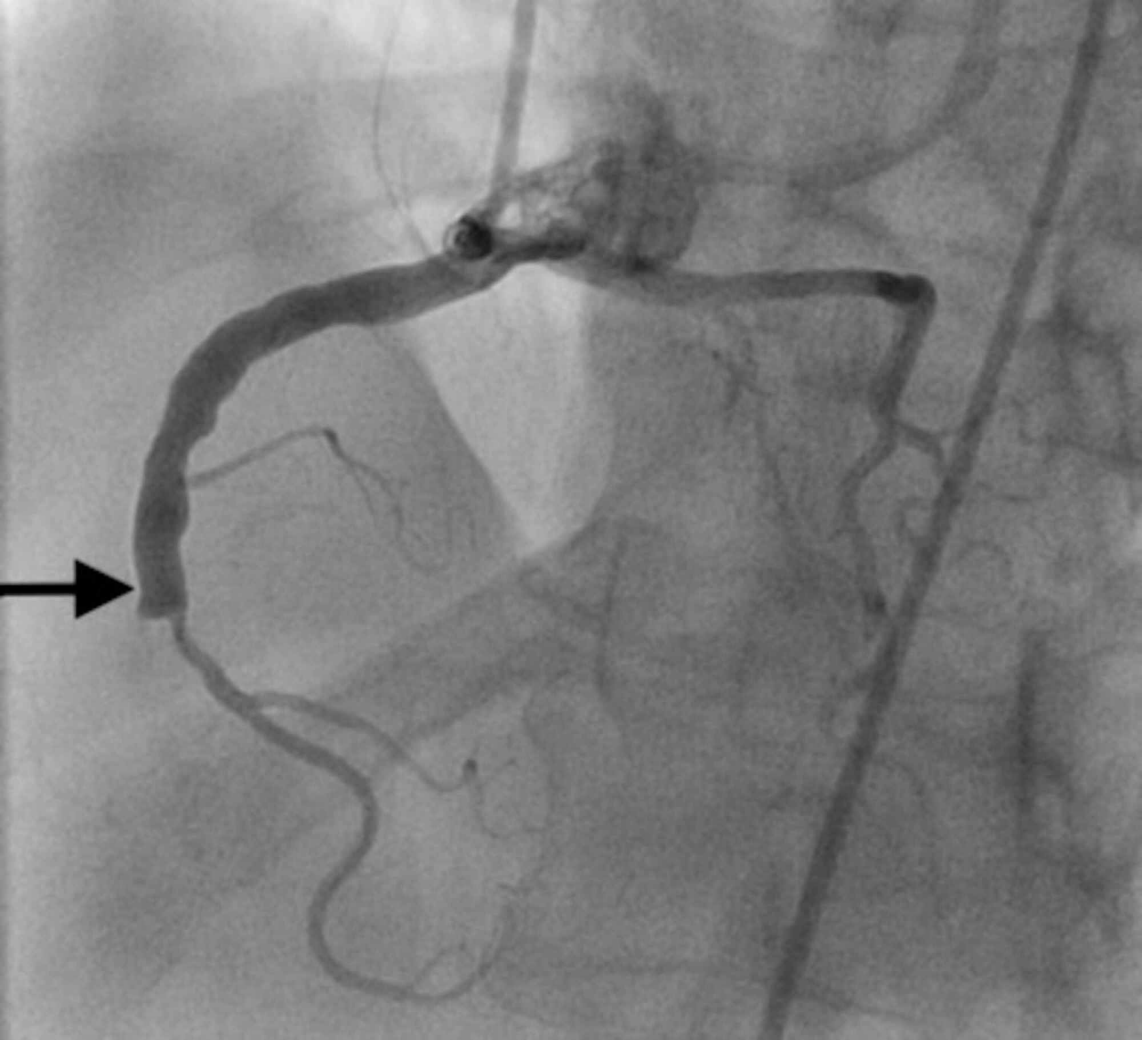
Selective coronary angiography of the right coronary artery revealing common origin of the RCA and LMCA from the RSV along with 100% mid occlusion of the RCA with TIMI-0 flow distally (arrow) RCA - right coronary cusp; LMCA - left main coronary artery; RSV - right sinus of Valsalva; TIMI - thrombolysis in myocardial infarction

**Figure 6:**
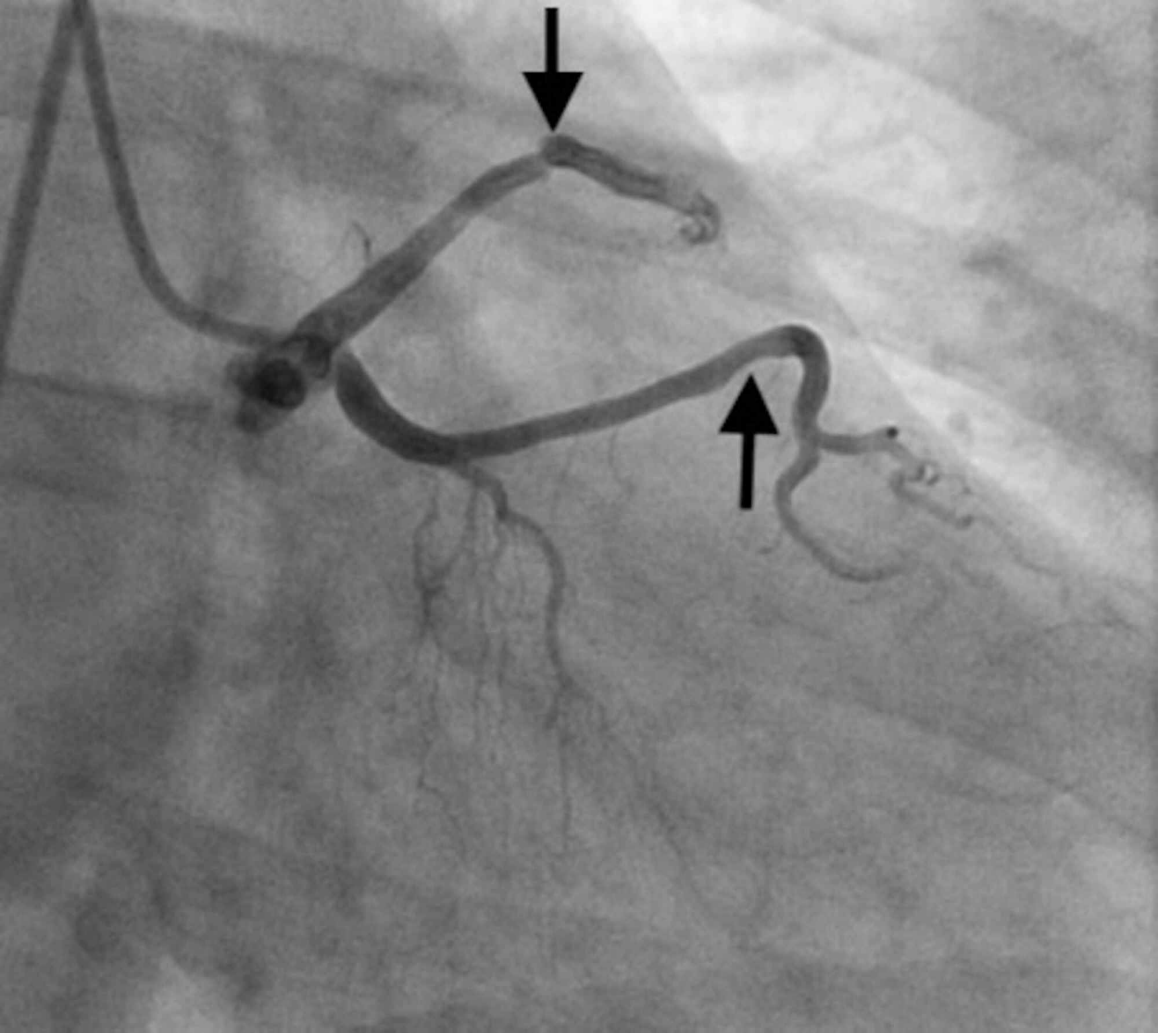
Selective angiography of the left main coronary artery showing a 90% stenosis in the mid LCX (down arrow) and a 50% stenosis in the mid LAD (upward arrow) with TIMI-3 flow LCX - left circumflex artery; LAD - left anterior descending; TIMI - thrombolysis in myocardial infarction

A 67-year-old Caucasian woman presented to ED with complaints of recurrent changing patterns of sub-sternal chest pressure radiating to the back and upper jaw. She had a history of chronic smoking. The initial ECG showed old inferior infarct and new T wave abnormality suggestive of ischemia. Coronary angiography was performed due to ongoing symptoms, which revealed an anomalous origin of LMCA from the right coronary cusp. There was 90% stenosis of the mid LCX and 50% stenosis in LAD. RCA had 100% occlusion in mid-segment with the faint left to right collaterals (Figures 5 and 6). CTS was consulted who recommended urgent CABG given high-grade stenosis in RCA and LCX. The patient underwent a triple vessel bypass with Left IMA to LAD, RIMA to obtuse marginal (OM), and SVG graft to RCA. She had an unremarkable postoperative hospital course and was discharged home in a stable condition. One year later, she presented with acute coronary syndrome/non-ST elevation myocardial infarction (NSTEMI). This time, her coronary angiography showed patent SVG-RCA graft with atretic left and right IMA grafts. The LCX was also 90% occluded, and she subsequently underwent successful percutaneous coronary intervention (PCI) with drug-eluting stent placement. Medical therapy was continued, and the patient continues to be symptom-free on subsequent follow-up visits.

## Discussion

The majority of congenital anomalies involving the origin of coronary arteries are benign and asymptomatic in 80% of the cases [1-3]. Based on its course, anomalous LMCA arising from the RCA has four sub-categories: inter-arterial where the LMCA courses between the aorta and pulmonary artery, intra-myocardial or intramural through the sub-endocardial or intra-myocardial region of ascending aorta, retro-aortic - posterior to the aortic root, and anterior - over the right ventricular outflow tract [4]. Of these, LMCA, with an initial intramural course in the wall of ascending aorta, carries the highest risk for sudden cardiac death [5]. The exact mechanism of coronary ischemia in these patients is not well understood. Several hypotheses include the compression of the smaller and slit-like ostium of LMCA, leading to loss of blood flow, superimposed vasospasm from endothelial dysfunction, or compression of the intra-mural segment due to hypertension and an ostial "ridge" which obstructs the blood flow during increased flow states [6].

The clinical presentation in patients with anomalous LMCA from RSV varies. On review of literature, only 20% of the patients present with symptoms mainly including exertional angina, dyspnea, or syncope [7-9]. Unfortunately, in the vast number of cases, sudden cardiac death is the initial and frequently terminal presentation [7]. Electrocardiographic findings in symptomatic patients are nonspecific and could vary from ST-wave abnormalities suggesting ischemia to ventricular tachycardia or fibrillation [10, 11]. An echocardiogram is typically obtained to assess left ventricular systolic function, but it does not provide any additional diagnostic information. Trans-esophageal echocardiography is usually not the best modality to delineate the origin and course of the coronary arteries, with the gold standard being a coronary angiogram [1, 12]. Coronary CTA may be used to rule out an intramural and inter-arterial course of the artery. 

Surgical intervention is a class 1 recommendation in patients with an established diagnosis. Different methods may be used [13-16]. The latest and most commonly used method nowadays is called “un-roofing” and involves an anterior aortotomy with incision of the common wall between the aorta and intramural segment of the anomalous coronary artery [14]. It recently gained traction as the procedure of choice for young patients with an intra-arterial or intramural malignant course [15, 16]. Potential pitfalls include damage to the intracoronary commissure, which may cause aortic insufficiency, exposure of layers of aortic wall to systemic pressure at the site of neo-ostium, which may create a localized dissection sometimes requiring repair or aggressive over roofing beyond the intramural segment which can result in aggressive bleeding.

Pulmonary artery translocation is the second method of surgical correction and may be used if the anomalous artery is not intramural and compressed between the great vessels [13]. This procedure involves mobilization of the main pulmonary root through meticulous dissection and moving its course, thus preventing it from compressing the anomalous coronary. An alternative version is dividing the right pulmonary artery at its origin, transposing it anterior to the aorta, and re anastomosis to the original site.

If the left and right coronary orifices are separated with little or no intramural segment, then re-implantation of ostia, which is a somewhat challenging technique, may be the best option. Risks include potential damage to aortic wall commissure, causing insufficiency [17].

The most technically challenging procedure is an "anatomical repair" which involves the creation of a neo-ostium for the anomalous vessel in the sinus from where it would normally have exited [13, 18]. In this procedure, an incision is made in the coronary sinus with the corresponding incision in the coronary artery away from the aorta. These incisions are then joined with augmentation of open areas with a patch. It may be used in all anatomical variants with or without an intramural course. Initial results are satisfactory, but long term outcomes need to be determined. Also, the surgical expertise required for this kind of procedure makes it less likely the procedure of choice in the future [18].

Standard coronary artery bypass grafting (CABG) with a saphenous or internal mammary vein grafting is an option used in many centers for rerouting blood flow around the intramural segments, however, performing a bypass graft without ligating the native vessel can lead to occlusion of the patent native vessels from competitive flow [19-20]. Using internal mammary arteries limits revascularization options for these patients in the future and because of competitive flow from native coronaries, puts them at risk for atrophy and disuse occlusion as happened in case 3. CABG should be limited to only those patients in which the anomaly is accompanied by significant atherosclerotic narrowing and where alternative procedures produced unsatisfactory results [13, 19-20].

Summarizing the above, surgical un-roofing is usually the procedure of choice in coronaries with intramural course whereas re-implantation or ostial reconstruction is used if there is little to no intramural course. In our first patient (Case 1), coronary angiography revealed LMCA from the right coronary cusp with a malignant course detected on coronary CTA. There was high-grade stenosis in RCA and LAD as well. In our second patient (Case 2), angiography revealed non-obstructive stenosis in the coronary arteries with a malignant course of LMCA from the right cusp, which required the relocation of the ostium. In our last patient (Case 3), the angiogram revealed a non-stenosed LMCA originating from RSV with the course of LCX posterior to the aorta. RCA, in this case, was completely occluded. She underwent a triple coronary artery bypass.

## Conclusions

Despite stenosis being non-significant in the coronary arteries per se, CABG was required in all three of our patients. It’s possible that the compression of LMCA between the aorta and pulmonary artery may have led to their presentation. LMCA from the right sinus of Valsalva is thus a rare but often fatal anomaly. Due to a lack of data and inability to predict the risk of sudden cardiac death, major society guidelines recommend surgical intervention (class 1 recommendation) for all patients regardless of ischemia or symptoms. 
